# The Effect of Krill Oil Supplementation on Exercise Performance and Markers of Immune Function

**DOI:** 10.1371/journal.pone.0139174

**Published:** 2015-09-25

**Authors:** Mariasole Da Boit, Ina Mastalurova, Goda Brazaite, Niall McGovern, Keith Thompson, Stuart Robert Gray

**Affiliations:** Institute of Medical Sciences, Foresterhill, University of Aberdeen, Aberdeen, Scotland, United Kingdom; University of Birmingham, UNITED KINGDOM

## Abstract

**Background:**

Krill oil is a rich source of the long-chain n-3 polyunsaturated fatty acids (PUFAs), eicosapentaenoic acid (EPA) and docosahexaenoic acid (DHA), which may alter immune function after exercise. The aim of the study was to determine the effects of krill oil supplementation on post exercise immune function and performance.

**Methods:**

Nineteen males and 18 females (age: 25.8 ± 5.3 years; mean ± S.D.) were randomly assigned to 2 g/day of krill oil (n = 18) or placebo (n = 19) supplementation for 6 weeks. A maximal incremental exercise test and cycling time trial (time to complete set amount of work) were performed pre-supplementation with the time trial repeated post-supplementation. Blood samples collected pre- and post- supplementation at rest, and immediately, 1 and 3h post-exercise. Plasma IL-6 and thiobarbituric acid reactive substances (TBARS) concentrations and, erythrocyte fatty acid composition were measured. Natural killer (NK) cell cytotoxic activity and peripheral blood mononuclear cell (PBMC) IL-2, IL-4, IL-10, IL-17 and IFNγ production were also measured.

**Results:**

No effects of gender were noted for any variable. PBMC IL-2 and NK cell cytotoxic activity were greater (P < 0.05) 3h post exercise in the krill oil compared to the control group. Plasma IL-6 and TBARS, PBMC IL-4, IL-10, IL-17 and IFNγ production, along with performance and physiological measures during exercise, were not different between groups.

**Conclusion:**

Six weeks of krill oil supplementation can increase PBMC IL-2 production and NK cell cytotoxic activity 3h post-exercise in both healthy young males and females. Krill oil does not modify exercise performance.

## Introduction

Fish oils are rich in the long chain n-3 polyunsaturated fatty acids (PUFAs) eicosapentaenoic acid (EPA) and docosahexaenoic acid (DHA), which have been found to have positive effects in chronic inflammatory disease, such as cardiovascular disease [1] and diabetes mellitus [2]. PUFAs are essential constituents of cell membranes of immune cells and are precursors of inflammatory mediators, such as prostaglandins and leukotrienes (for review see [3]). The primary PUFA found in cell membranes of leukocytes is the n-6 PUFA arachidonic acid (AA). However, increasing dietary consumption of EPA and DHA will alter the cell membrane composition and increase EPA and DHA levels with a concomitant reduction in the amount of AA [4]. This alteration in fatty acid composition alters the substrate used for eicosanoid synthesis, resulting in a reduction in pro-inflammatory AA-derived mediators and an increase in the less potent EPA/DHA-derived alternative inflammatory mediators, such as the 3-, rather than the 2-, series prostaglandins [5]. Fish oils are not the only source of EPA and DHA with Antarctic krill also being a rich source of n-3 PUFAs. The primary difference between fish and krill oil is that while fish oil contains the majority of n-3 PUFAs as triacylglycerol (TG) krill oil consists of more than 40% of n-3 PUFAs as phospholipids (PL) [6]. Krill oil also contains the antioxidant astaxanthin. During supplementation studies, similar increases in plasma EPA and DHA can be seen when a third less PUFAs are given as krill compared to fish oil supplements [7]. In another study when similar amounts of fish oil and krill oil, 600mg/day, are given the increases in plasma and erythrocyte EPA and DHA were found to be greater with krill compared to fish oil [8].

Fish oil supplements have been recommend for athletic populations due to evidence of their anti-inflammatory, antithrombotic, antiarrhythmic, hypolipidemic and antiproliferative properties. It is thus suggested that the majority of athletes, especially those at leisure level, should consume 1–2 g/day of EPA and DHA to prevent muscle and joint inflammation and improve overall health. Over time this would not only improve overall health but may ultimately improve performance [9]. In spite of these assertions the evidence to support the use of n-3 PUFAs specifically in exercising populations is limited and there is also concern that due to their high number of double bonds an increase in n-3 PUFA consumption may result in an increase in lipid peroxidation.

Whilst both fish and krill oil are rich n-3 PUFAs the majority of previous research in this area has utilised fish oil supplementation. One area where n-3 PUFA supplements may be useful is in the post-exercise recovery period where there are a variety of perturbations in the immune system [10]. However there are a few studies which have investigated the effects of fish oil supplementation on various aspects of immune function after exercise. Toft and colleagues found that fish oil supplementation had no effect on plasma cytokine levels after exercise [11]. One study found higher *ex vivo* activated peripheral blood mononuclear cell proliferation (PBMC) [12]. In recent work we demonstrated that fish oil supplementation results in an increase in PBMC IL-2 production and natural killer (NK) cell cytotoxic activity in the recovery period after exercise in healthy young males [13]. Whether this effect will be seen in females remains to be established. It has also been suggested that, via different mechanisms, n-3 PUFA supplementation may improve exercise performance. Indeed n-3 PUFA supplementation has been found to reduce HR and $\dot{\text{V}}{\text{O}}_{2}$ during exercise [14–17], although our findings do not support this [13]. Furthermore these cardiometabolic changes do not translate into improvements in exercise performance [14, 16, 18], although one study did find an increase in $\dot{\text{V}}{\text{O}}_{2\text{max}}$ after fish oil supplementation [19]. The effects of krill oil on cardiometabolic variable and exercise performance remain to be established.

The aim of the present study was to investigate the effects of six weeks of supplementation with krill oil on markers of immune function (NK cell cytotoxicity and PBMC Th1/Th2 cytokine production) following an acute bout of endurance exercise in both males and females. A secondary aim was to determine whether krill oil supplementation altered exercise performance during a simulated cycling time trial.

## Methods

### Subjects

Nineteen males and 18 females (aged 25.8 ± 5.3 years, height 175 ± 11.9 cm, weight 72.5 ± 14.9 kg; mean ± S.D.) volunteered to participate in the study. The study was approved by the University of Aberdeen College of Life Sciences and Medicine Ethics Review Board and participants were made aware of the aims, risks and potential discomfort associated with the study before providing written informed consent.

### Supplementation

Participants were randomly assigned to either a placebo (n = 19) or krill oil (n = 18) group. Capsules were closely matched for both colour and shape, and both participants and investigators were blind to the supplementation group. After the preliminary maximal exercise test, participants in the placebo group consumed 2g (4*500mg) of placebo oil daily, while those in the krill oil group consumed 2g (4*500mg) of krill oil daily for a six week period. All capsules were provided by Aker Biomarine Antarctic AS (Oslo, Norway). The placebo oil was produced to reflect the fatty acid composition of the average European diet [20]. Each 500mg krill oil capsule contained 60mg EPA, 30mg DHA and 61μg astaxanthin. To put this into a dietary context a 140g portion of oily fish would typically provide approximately 2.8 g EPA plus DHA, although this is known to vary dramatically between wild and farmed fish due to recent changes in feeds [21].

### Maximal Exercise Test

For the 24h period prior to each visit, participants were instructed to refrain from the consumption of alcohol, caffeine and strenuous exercise. For the same period before the maximal exercise test, participants were asked to record their dietary intake and replicate this prior to the post-supplementation trial. All tests began at the same time of day, between 7 and 10am. A fasting blood sample was collected before performing an incremental maximal exercise test on a cycle ergometer (Lode, Netherlands). Participants cycled at self-selected cadence (70–90 rpm) with workload increasing by 30 Watts every minute for males and 20 Watts every minute for females until volitional exhaustion. Gas exchange (Medical Graphics, UK) and heart rate (Polar, Finland) were monitored throughout the test. $\dot{\text{V}}{\text{O}}_{2\text{peak}}$ was taken as the highest $\dot{\text{V}}{\text{O}}_{2}$ measured over a 30 second period and a workload estimated to elicit 70% $\dot{\text{V}}{\text{O}}_{2\text{peak}}$ calculated to be used in the exercise trials.

### Baseline Exercise Trial

The exercise trial was a simulated time trial with the ergometer set in the linear mode. Approximately 7 days prior to this trial a familiarisation trial was carried out where no physiological measures were made but the participant completed the time trial as below. After a 5 min warm up at 75 Watts subjects performed a set amount of work in as quick a time as possible. The amount of work was based on the results of the maximal exercise test as:

Work = 0.70*Wmax*3600

And the linear factor (L) used to set up the ergometer was calculated as:

W = L*(rpm)2

This factor was chosen so that 70% of Wmax would be achieved at around 80rpm which is the preferred pedal rate, in our experience, in this population. During the time trial participants did not receive any information on workload, pedal rate, time or heart rate with only the amount of work completed and the amount remaining passed on to participants. Heart rate and gas exchange (3 min recording) were recorded at approximately 25, 50 and 75% of the percentage of work performed. Water was available *ad libitum*. This trial was the same as the post-supplementation trial, except no blood samples were collected during this trial.

### Post-supplementation Trial

After the 6-week supplementation period, fasted blood samples were taken. Participants then performed the time trial as in the baseline exercise trial. Immediately on cessation of exercise a post-exercise blood sample was drawn. During a 3h recovery period, participants remained fasted, and further blood samples were taken 1h and 3h post-exercise. Throughout the post-supplementation trial heart rate and gas exchange were recorded in the same way as during the baseline exercise trial. Participants were permitted to consume water *ad libitum*.

### Blood Sampling

Blood samples were drawn from an antecubital vein using a butterfly needle (21G). Blood was collected in vacutainers coated with Lithium-Heparin (BD, Oxford, UK), for peripheral blood mononuclear cell (PBMC) isolation, and K^+^EDTA (BD, Oxford, UK) for separation of plasma. The K^+^EDTA vacutainer was centrifuged at 800g for 10 minutes at 4°C and plasma stored at -80°C until analysis.

### Erythrocyte Fatty Acid Composition Analysis

Fatty acid analysis was performed in EDTA blood samples sent to a commercial laboratory (Omegametrix) and analysed as previously described [22]. Samples were collected in 4 ml EDTA vacutainers, mixed and immediately shipped to Germany for analysis. Briefly, fatty acid methyl esters were generated from erythrocytes by acid transesterification and analysed by gas chromatography using a GC2010 Gas Chromatograph (Shimadzu, Duisburg, Germany) equipped with a SP2560, 100-m column (Supelco, Bellefonte, PA) using hydrogen as the carrier gas. Fatty acids were identified by comparison with a standard mixture of fatty acids characteristic of erythrocytes. A total of 26 fatty acids were identified and quantified. Results are given as percentage of total identified fatty acids after response factor correction. The coefficient of variation for EPA plus DHA and for most other fatty acids was ~4%. Analyses were quality-controlled according to DIN ISO 15189.

### Plasma IL-6

Measurements were performed using commercially available high sensitivity ELISA kits (R&D Systems, UK) according to the manufacturer’s instructions.

### Plasma thiobarbituric acid reactive substances (TBARS)

Measurements were performed using commercially available parameter assay kits (R&D Systems, UK) according to the manufacturer’s instructions. The principle of this assay is that malondialdehyde (MDA), from lipid peroxides, in the presence of heat and acid, reacts with 2-thiobarbituric acid to produce a coloured end product which can be measured in a spectrophotometric plate reader. For this reason TBARS results are expressed as MDA concentration.

### PBMC Isolation and Incubation

These procedures were carried out using the same methods as our previous work [13] where the protocol was sufficient to show a robust stimulation of cytokine production. Twenty ml whole blood was mixed with 10ml PBS and layered onto 20ml Histopaque (Sigma-Aldrich, St Louis, USA) before being centrifuged at 400g for 30 minutes at room temperature. The PBMC layer was collected and mixed with 40ml PBS and centrifuged at 250g for 10 minutes. The supernatant was then removed and the cell pellet reconstituted with 20ml PBS. This washing phase was then repeated. The final pellet was reconstituted with 1ml RPMI-1640 medium and cell numbers determined. 1.5x10^6^ cells, in duplicate, were then incubated at 37°C and 5% CO_2_ for 24h supplemented with 2mM glutamine, antibiotics, 2.5% autologous plasma and 10 mg/L concanavalin A (Con A). At the end of incubation, each well was centrifuged at 250g for 10 minutes, the supernatant removed and frozen at -80°C for analysis of PBMC cytokine production.

### PBMC Cytokine Production

All measurements were performed using commercially available multiplex assay kits (R&D Systems, UK) according to the manufacturer’s instructions (Luminex, USA). The cytokines IL-2, IL-4, IL-10, IL-17 and IFN-γ were measured in culture supernatants.

### Natural Killer Cell Cytotoxic Activity

Natural killer cell cytotoxic activity was measured in PBMCs, isolated as above but prior to treatment/incubation, using NKTEST kits (Opregen Pharma, Heidelberg, Germany) according to the manufacturer’s instructions. While this kit will measure primarily NK cell cytotoxic activity other cell types with NK cell-like activity, ie γδ T cells, will make a minor contribution. Briefly, PBMCs were mixed with pre-stained K562 target cells in ratios of 50:1 and 25:1. Tubes were centrifuged for 3 min at 120g and then incubated for 2h in a CO_2_ incubator at 37°C. 50μl of DNA staining solution was added to the cells, which were incubated in the dark on ice for 5 min before analysis by flow cytometry. 2,500 cells were analysed for each sample, allowing us to determine the effects of exercise and krill oil. To discriminate target and effector cells a gate was set in the green fluorescence histogram. The percentage of dead cells was then determined using the red fluorescence histogram and the percentage specific cytotoxicity determined by subtracting the percentage dead cells in a tube containing K562 cells alone from the percentage of dead target cells in the samples.

### Statistical Analysis

Data analysis was carried out using SPSS software. Baseline characteristic data were compared between groups using independent t-tests. Initial analyses by sex found no effects and therefore are not presented here. Data were then analysed using a two way (time and group) repeated measures ANOVA with Bonferroni corrected post-hoc t-tests performed where appropriate. Statistical significance was accepted at P <0.05. All data are expressed as mean ± SD.

## Results

### Baseline characteristics

Subject baseline characteristics are summarised in Table 1. Baseline physical characteristics were similar between the groups.

**Table 1:** Baseline characteristics of subjects in control and krill oil groups. Values are mean ± SD.

Measure	Control Group (n = 19: 10 females)	Krill Oil Group (n = 18: 8 females)	P Value
Age (years)	26 ± 6	25 ± 5	0.757
Height (cm)	176 ± 12	175 ± 12	0.670
Weight (kg)	73.5 ± 16.4	71.3 ± 13.7	0.318
$\dot{\text{V}}{\text{O}}_{2\text{max}}$ (ml/kg/min)	41.6 ± 7.8	43.6 ± 6.4	0.439

### Erythrocyte fatty acid composition

There were no differences in erythrocyte fatty acid composition between groups at baseline. In the control group there were no changes in erythrocyte fatty acid composition over the supplementation period. However in the krill oil group there were significant increases (P<0.05) in levels of C20:5n3, C22:6n3 and the Omega-3 index, with decreases (P<0.05) in levels of C20:4n6 and C22:4n6 (Table 2).

**Table 2:** Erythrocyte fatty acid composition in control and krill oil groups at rest pre- and post-supplementation (6 weeks). Values are mean ± SD. * indicates a significant difference (P<0.05) from baseline values. (c = cis and t = trans)

	Control Group (n = 19: 10 female)		Krill Oil Group (n = 18: 8 females)	
Fatty Acid	Pre-Supplementation	Post-Supplementation	Pre-Supplementation	Post-Supplementation
**C14:0**	0.35 ± 0.10	0.39 ± 0.13	0.36 ± 0.11	0.39 ± 0.13
**C16:0**	21.85 ± 0.92	21.81 ± 0.95	21.91 ± 0.88	22.37 ± 1.01
**C16:1n7t**	0.12 ± 0.03	0.15 ± 0.06	0.12 ± 0.02	0.13 ± 0.04
**C16:1n7**	0.34 ± 0.12	0.36 ± 0.16	0.30 ± 0.12	0.29 ± 0.12
**C18:0**	16.46 ± 0.98	16.13 ± 1.52	16.34 ± 1.05	16.13 ± 0.86
**C18:1t**	0.40 ± 0.28	0.36 ± 0.10	0.30 ± 0.10	0.32 ± 0.08
**C18:1n9**	16.06 ± 0.86	15.77 ± 1.11	16.39 ± 0.85	16.29 ± 1.57
**C18:2n6tt**	0.11 ± 0.05	0.10 ± 0.05	0.12 ± 0.05	0.10 ± 0.05
**C18:2n6ct**	0.05 ± 0.04	0.03 ± 0.02	0.03 ± 0.02	0.04 ± 0.03
**C18:2n6tc**	0.13 ± 0.06	0.12 ± 0.08	0.17 ± 0.15	0.10 ± 0.07
**C18:2n6**	12.87 ± 2.20	13.37 ± 2.16	13.77 ± 1.55	13.80 ± 1.75
**C20:0**	0.15 ± 0.04	0.15 ± 0.03	0.15 ± 0.04	0.16 ± 0.04
**C18:3n6**	0.08 ± 0.02	0.10 ± 0.06	0.07 ± 0.04	0.08 ± 0.02
**C20:1n9**	0.27 ± 0.06	0.28 ± 0.13	0.29 ± 0.07	0.29 ± 0.05
**C18:3n3**	0.17 ± 0.06	0.19 ± 0.10	0.20 ± 0.05	0.24 ± 0.09
**C20:2n6**	0.24 ± 0.06	0.24 ± 0.04	0.23 ± 0.05	0.25 ± 0.05
**C22:0**	0.32 ± 0.17	0.40 ± 0.18	0.33 ± 0.13	0.33 ± 0.12
**C20:3n6**	1.91 ± 0.50	1.93 ± 0.50	1.82 ± 0.38	1.75 ± 0.36
**C20:4n6**	14.93 ± 1.42	14.44 ± 1.36	14.50 ± 1.56	13.45 ± 1.11*
**C24:0**	0.85 ± 0.38	0.90 ± 0.53	0.80 ± 0.26	0.76 ± 0.37
**C20:5n3**	0.74 ± 0.34	0.80 ± 0.19	0.82 ± 0.34	1.48 ± 0.73*
**C24:1n9**	0.86 ± 0.23	0.81 ± 0.15	0.82 ± 0.29	0.79 ± 0.22
**C22:4n6**	2.99 ± 0.50	2.87 ± 0.52	2.72 ± 0.57	2.27 ± 0.46*
**C22:5n6**	0.61 ± 0.16	0.62 ± 0.15	0.49 ± 0.14	0.45 ± 0.13
**C22:5n3**	2.40 ± 0.37	2.31 ± 0.32	2.44 ± 0.41	2.54 ± 0.32
**C22:6n3**	4.54 ± 1.06	4.60 ± 1.09	4.49 ± 1.08	5.43 ± 1.24*
**Omega-3 Index**	5.20 ± 1.28	5.40 ± 1.19	5.32 ± 1.36	6.79 ± 1.66*

### Time trial variables

**Fig 1:**
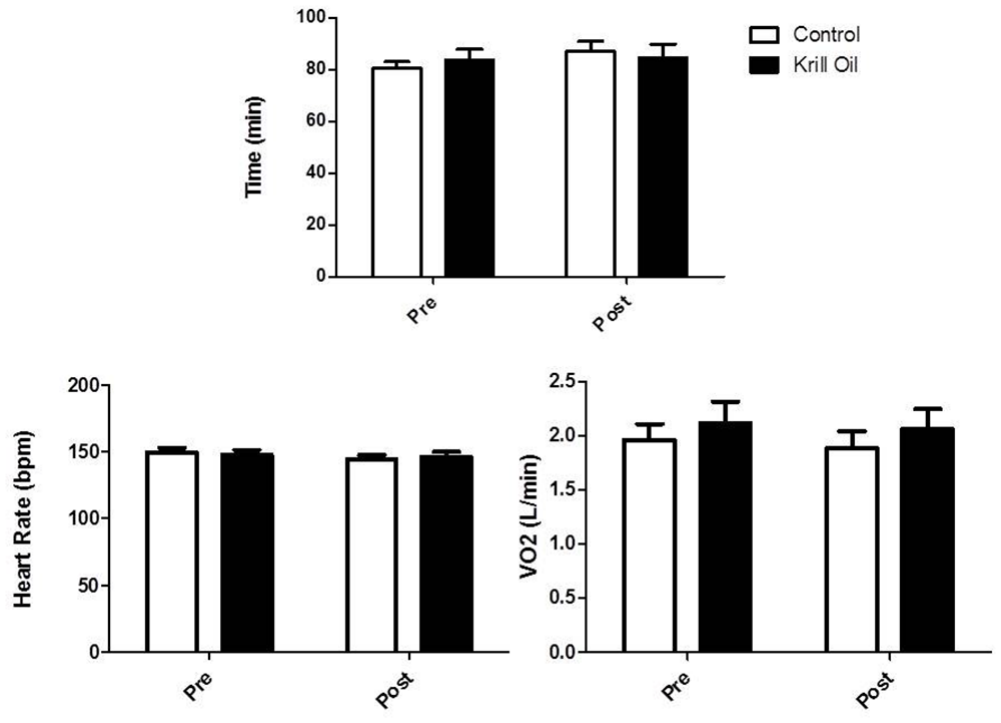
The effect of 6 weeks krill oil/placebo supplementation on heart rate, oxygen consumption and time taken to complete the time trial (pre and post 6 weeks supplementation).

The ANOVA revealed no time (F_(3,105)_ = 3.67, P = 0.07), group (F_(1,35)_ = 0.005, P = 0.94) or interaction (F_(3,105)_ = 2.42, P = 0.12) effect for time to completion during the time trial. In the control group the time to complete the baseline time trial was 80.5 ± 11.2 min and 84.7 ± 17.1 min after the supplementation period. In the krill oil group baseline time to complete was 83.9 ± 14.7 min and 85.4 ± 19.8 min after the supplementation period. Similarly no time (HR: F_(3,105)_ = 3.01, P = 0.09; $\dot{\text{V}}{\text{O}}_{2}\;F_{\left(3,105\right)}=\;3.52$, P = 0.07), group (HR: F_(1,35)_ = 0.001, P = 0.97. $\dot{\text{V}}{\text{O}}_{2}\;F_{\left(1,35\right)}=\;0.48$, P = 0.50), or interaction (HR: F_(3,105)_ = 1.22, P = 0.28. $\dot{\text{V}}{\text{O}}_{2}\;F_{\left(3,105\right)}=\;0.08$, P = 0.78) effects were noted for HR or $\dot{\text{V}}{\text{O}}_{2}$ during the time trial (Fig 1).

### Plasma IL-6 and TBARS

**Fig 2:**
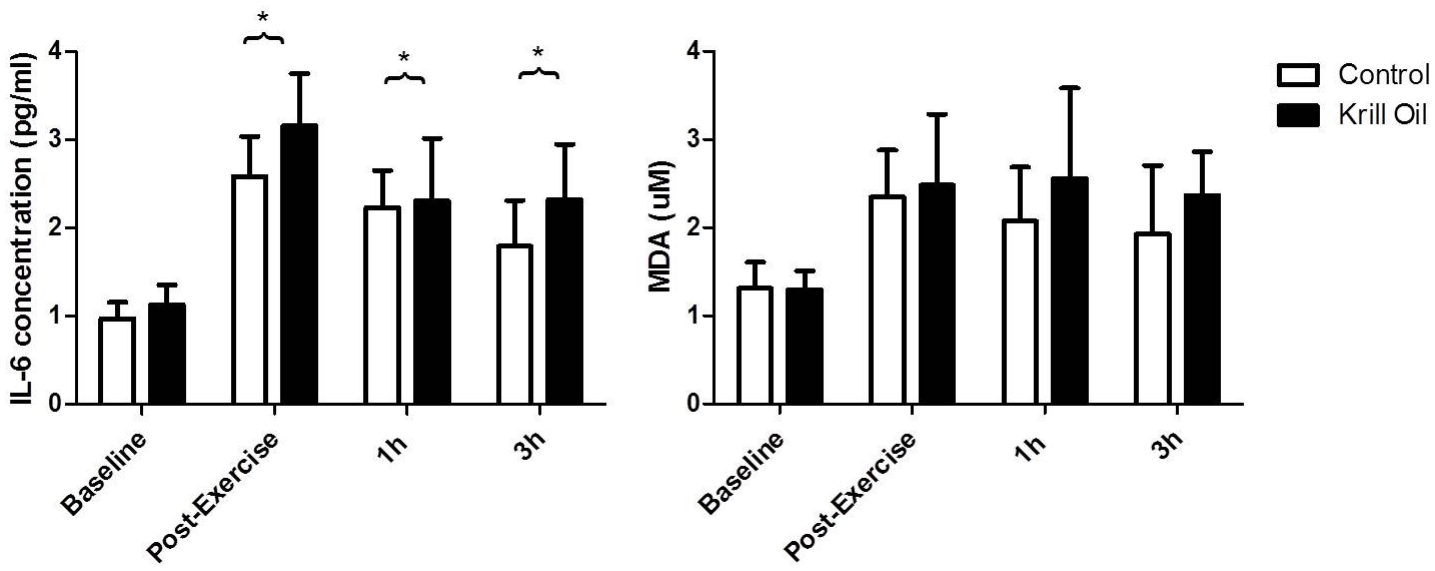
The effect of 6 weeks krill oil/placebo supplementation on plasma IL-6 and TBARS (expressed as MDA concentration) before and after exercise. * denotes a significant difference (P<0.05) from baseline in both groups.

The ANOVA for plasma IL-6 showed a time (F_(3,105)_ = 7.6, P<0.05) effect with no group (F_(1,35)_ = 0.45, P = 0.50) or interaction (F_(3,105)_ = 0.40, P = 0.75) effects. Plasma IL-6 was higher (P<0.05) immediately, 1h and 3h post-exercise, compared to baseline, in both groups. There were no time (F_(3,105)_ = 2.13, P = 0.10), group (F_(1,35)_ = 0.15, P = 0.71) or interaction (F_(3,105)_ = 0.12, P = 0.95) effects for plasma TBARS (Fig 2).

### PBMC Cytokine Production

**Fig 3:**
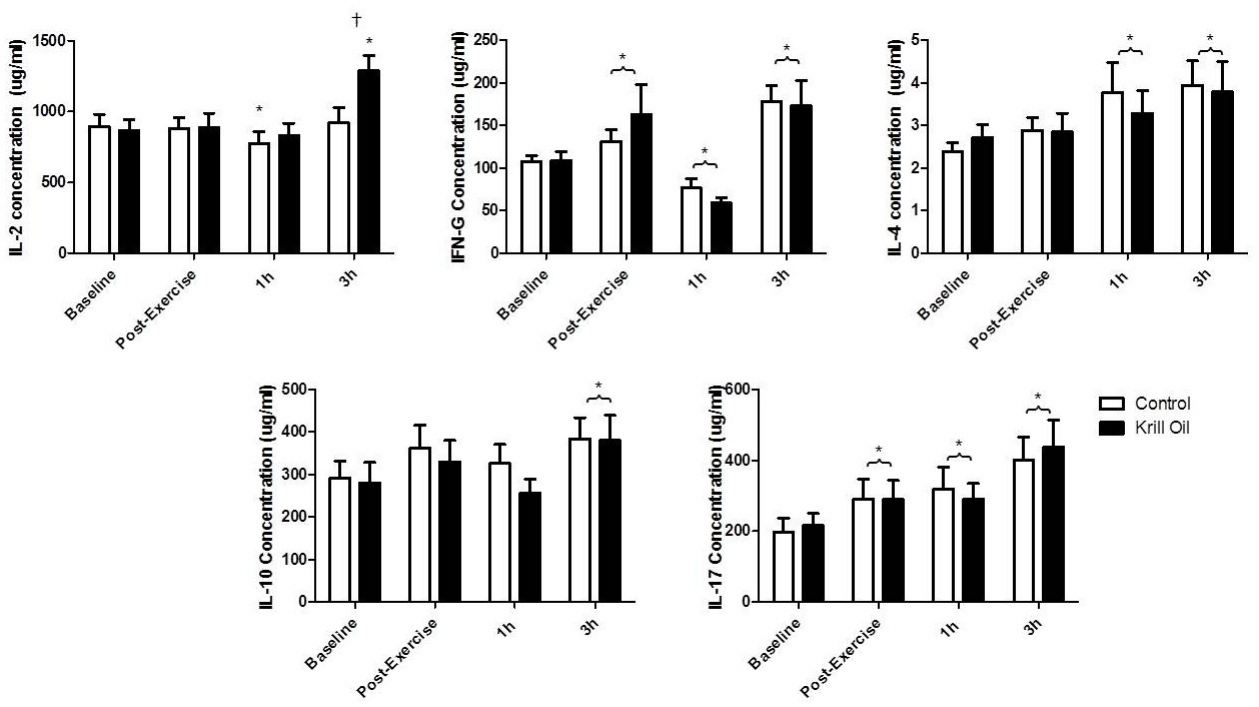
The effect of 6 weeks krill oil/placebo supplementation on exercise induced cytokine production by PBMCs stimulated with concanavalin A. * indicates significant difference (P<0.05) from baseline values. † indicates significant difference (P<0.05) between groups.

PBMC cytokine data is shown in Fig 3. The ANOVA revealed an interaction (F_(3,105)_ = 3.17, P = 0.02) and time effect (F_(3,105)_ = 6.43, P<0.05) for PBMC IL-2 production. Post-hoc analysis revealed that PBMC IL-2 was lower (P<0.05) than baseline, at 1h post-exercise in the control group and higher (P<0.05) than baseline, at 3h post-exercise in the krill oil group. Furthermore 3h post-exercise PBMC IL-2 was higher (P<0.05) in the krill oil compared to the control group. No group effect (F_(1,35)_ = 1.11, P = 0.30) was observed for PBMC IL-2. Analysis showed an effect of time for PBMC IFN-γ (F_(3,105)_ = 18.54, P<0.05) with post-hoc analysis revealing that PBMC IFN-γ was higher (P<0.05) immediately and after 3h, and lower at 1h, post-exercise compared with baseline in both groups. No group (F_(1,35)_ = 0.02, P = 0.89) or interaction (F_(3,105)_ = 0.94, P = 0.42) effect was observed for PBMC IFN-γ. The ANOVA revealed a time effect for PBMC IL-4 (F_(3,105)_ = 3.27, P<0.05) with post hoc analysis showing that PBMC IL-4 was greater (P<0.05) at 1h and 3h post-exercise, compared with baseline, in the two groups. The analysis of PBMC IL-4 showed no interaction (F_(3,105)_ = 0.26, P = 0.85) or group effect (F_(1,35)_ = 0.04, P = 0.84). Analysis of PBMC IL-10 found an effect of time (F_(3,105)_ = 5.763, P<0.05) with post-hoc analysis revealing that PBMC IL-10 was higher (P<0.05) 3h post-exercise compared with baseline in both groups. No group (F_(1,35)_ = 0.24, P = 0.63) or interaction (F_(3,105)_ = 0.59, P = 0.62) effect was observed for PBMC IL-10. The ANOVA revealed a time effect for PBMC IL-17 (F_(3,105)_ = 12.86, P<0.05) with post-hoc analysis revealing that PBMC IL-17 was higher (P<0.05) at every time point compared with baseline, in both groups. No group (F_(1,35)_ = 0.01, P = 0.91) or interaction (F_(3,105)_ = 0.32, P = 0.81) effect was observed for PBMC IL-17.

### NK cell function

**Fig 4:**
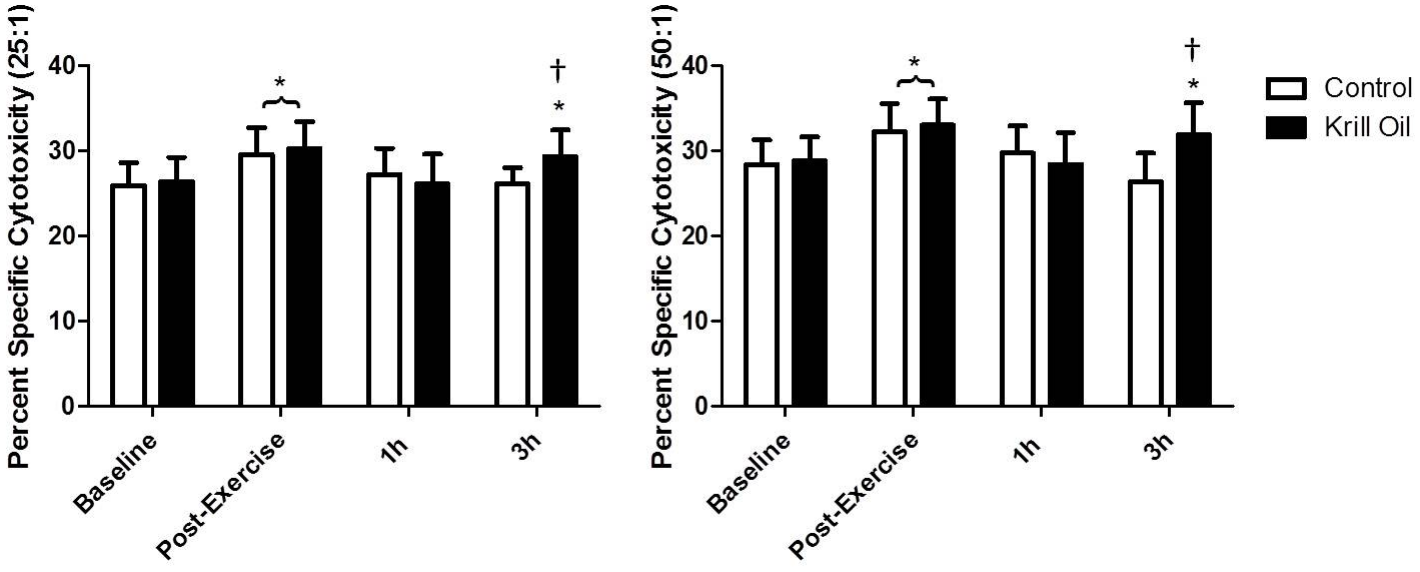
The effect of 6 weeks krill oil/placebo supplementation on NK cell cytotoxic activity at 50:1 and 25:1 effector:target cell ratio, before and after exercise. * indicates significant difference (P<0.05) from baseline values. † indicates significant difference between groups.

Analysis of natural killer cell cytotoxic activity revealed a significant interaction effect at both 50:1 (F_(3,105)_ = 8.18, P<0.05) and 25:1 (F_(3,105)_ = 4.09, P<0.05) effector:target cell ratios. Post hoc analysis revealed that at 3h post-exercise NK cell cytotoxic activity was greater in the krill oil group, compared to the control group, for both effector:target ratios of 50:1 and 25:1 (P<0.05 Fig 4). In addition, time effects in NK cell cytotoxic activity at the 50:1(F_(3,105)_ = 12.91, P<0.05) and 25:1 (F_(3,105)_ = 13.77, P<0.05) ratios were observed. Post-hoc analysis found that NK cell cytotoxic activity was greater (P<0.05) at 1h post-exercise, compared to baseline, in the two groups and higher at 3h post-exercise, compared to baseline, in the krill oil group only. There was no effect of group for NK cell cytotoxic activity at either ratio (50:1−F_(1,35)_ = 3.53, P = 0.07. 25:1−F_(1,35)_ = 1.51, P = 0.22).

## Discussion

The current study has demonstrated that supplementation of the diet with 2 g per day of krill oil for 6 weeks increases the production of IL-2 in stimulated PBMCs and the cytotoxic activity of NK cells in the recovery period after exercise. These effects were seen in both males and females. There was no effect of krill oil supplementation on plasma levels of IL-6 and TBARS, or on PBMC production of IL-4, IL-10, IL-17 and IFNγ in response to exercise. Similarly, krill oil supplementation did not alter time trial performance or the oxygen cost and HR response to exercise.

The current study has demonstrated that 6 weeks of supplementation with krill oil results in a 75% increase in erythrocyte EPA, a 21% increase in DHA and a 27% increase in the Omega-3 Index. Alongside these increases in n-3 PUFA there were concomitant decreases of 7% and 17% in levels of arachidonic and docosatetraenoic acid, respectively. Therefore, our study is in agreement with previous work [7] which has demonstrated that krill oil supplementation at a level of around 1/3rd lower than previous work with fish oil can result in a similar magnitude of a rise in EPA and DHA. It is worth pointing out here that the current study made no direct comparison to a group of participants supplemented with fish oil. However, due to the lack of studies in this area with krill oil supplementation we have compared our studies to those where fish oil supplementation was given. That is not to say that fish and krill oil are the exact same, as highlighted in the introduction.

There were no effects of krill oil supplementation on any measures of immune function and lipid peroxidation at rest in the current study, which is in agreement with previous work with fish oil supplementation in healthy populations [4, 13, 23]. Others have shown that supplementation with increasing quantities of EPA tends to result in an increase in NK cell cytotoxic activity, although PBMC IL-2 production was not altered, in healthy young males [24]. However, there are several studies in disagreement with this. In healthy older (>55y) participants it has been shown that 1.2 g/day EPA supplementation for 12 weeks can reduce NK cell cytotoxic activity [25]. Similar findings were observed in young healthy men who were supplemented with 6g/day DHA for 90 days [26]. Furthermore, *in vitro* studies of human NK cells and in young mice have found that EPA and DHA treatment reduces NK cell cytotoxic activity [27–29].

The current study has demonstrated that NK cell cytotoxic activity and PBMC IL-2, but not other cytokines, are increased after krill oil supplementation. What remains to be established are the mechanisms through which n-3 PUFA supplementation results in these alterations. It is known that PGE-2 can reduce the cytotoxic activity of human NK cells [30] and it is likely that the increase in EPA and DHA incorporation into immune cell membranes as a result of krill and fish oil supplementation will result in a concomitant decrease in membrane AA levels and thus altered eicosanoid synthesis [31]. Primarily this would reduce PGE-2 synthesis and relieve its suppressive effects on NK cells, although why we do not then see a universal increase in NK cell activity with fish oil supplementation, in the above studies, is not clear. What is clear, from our work, is that both krill and fish oil supplementation results in an increase in PBMC IL-2 production and NK cell cytotoxic activity and as exercise increases PGE-2 production this may explain why we only see these positive effects with exercise. Indeed when indomethacin, which inhibits PGE-2 production, is administered during exercise a greater NK cell cytotoxic activity is observed [32]. The current study has also found that there was no effect of krill oil on plasma IL-6 levels, which is in agreement with previous fish oil studies in this area [11, 13, 33].

There has been one study investigating the effects of krill oil supplementation on markers of oxidative stress and inflammation which, contrary to the current findings, demonstrated that in the recovery period after a bout of exercise, TBARS levels were lower in the krill oil group, compared to placebo [34]. No differences in tumor-necrosis factor-alpha (TNF-α) or the activity of superoxide dismutase, glutathione peroxidase and creatine kinase were observed between groups. Previous work which has investigated the effects of fish oil supplementation on markers of oxidative stress is equivocal. Work by Bloomer and colleagues found that fish oil supplementation had no effect on plasma TBARS after 1h of treadmill exercise [35], which is in agreement with the current findings. However, it has also been shown that fish oil supplementation increase plasma F2-isoprostanes following three days of 3h cycling sessions [36], but plasma TBARS were found to be reduced after eccentric knee extensor exercise [23]. It would appear therefore that EPA and DHA have no effect on markers of oxidative stress after acute submaximal exercise, but can reduce and increase such markers after acute eccentric and short-term intensified training, respectively.

The current data found that krill oil did not affect time trial performance, nor were heart rate or oxygen consumption affected. The fact that performance was not affected is in broad agreement with data published with fish oil where several studies have found no performance benefit, although cardiometabolic changes (ie reduced HR and $\dot{\text{V}}{\text{O}}_{2}$) were observed [14–17]. It is not clear why in the current study, and in our previous fish oil work, we have found no effect of n-3 PUFA supplementation on HR and $\dot{\text{V}}{\text{O}}_{2}$. The human studies where these effects have been observed have in general been at either lower (i.e. 55% peak workload—compared to ~70% $\dot{\text{V}}{\text{O}}_{2\text{max}}$ in the current study) or higher intensities (~10 min time trial to exhaustion) but it is not obvious why this would alter any effect of fish oil on these parameters. To clarify this it would be important to examine the effects of n-3PUFAs on HR and $\dot{\text{V}}{\text{O}}_{2}$ at varying exercise intensities within the same population. Together our data and that of others indicates that supplementation with n-3 PUFAs, either via fish or krill oil, does not alter exercise performance in young healthy volunteers. This lack of change in exercise performance may be due to the dose of krill, in the current study, and fish oil in previous studies, given not being sufficient to alter the fatty acid composition of muscle itself. Whilst we based our dose of krill oil on previous work comparing krill and fish oil [7] we have no data to show that this alter the muscle fatty acid composition. As measures of immune function were the primary outcome of the current study we chose not to collect muscle samples for such analysis but subsequent studies with a focus on exercise performance may wish to consider this. Furthermore it may also be the case that fish oil may still be of benefit for exercise performance in different populations, ie older people, or for different forms of exercise, ie strength training [37, 38]. On the other hand it may be, as the evidence suggests, that increasing n-3 PUFA consumption simply does not alter endurance exercise performance.

On top of the effects of krill oil supplementation on markers of immune function the current study also provides important information regarding the effects of exercise alone. Prior to discussion of these results, however, it is worth noting that we did not have a control group with no-exercise and so it is possible that these changes are circadian in origin. Our work would indicatively support recent findings that exercise results in an increase in the production of the T helper cell Th17 cytokine IL-17 [39], which has recently been identified in having an important role in the regulation of inflammation with precise roles still being identified [40]. The precise role of this increase in PBMC IL-17 during exercise is yet to be established and further work is needed in this area. IL-17 is known to drive the production of IL-6 and PGE-2 in many cell types, such as epithelial, endothelial, and fibroblastic cells [41] and can also stimulate T cell proliferation and IL-2 production [42], indicating several areas where IL-17 may have important roles during exercise. Furthermore determining the cell type that is producing IL-17 and whether we are observing and increase in individual cell IL-17 production or just a greater number of cells, e.g. Th17 cells, producing IL-17. We have also extended the pioneering work of Ostrowski and colleagues, who found increases in plasma IL-10 after exercise [43], by showing that exercise increases PBMC IL-10 production. We have further added to basic exercise immunology research by showing that these changes are seen in both males and females. In fact the majority of previous research in exercise immunology has been in male volunteers and so the current study is the first to find that the effects of such exercise on selective markers of immune function are independent of gender.

In conclusion, the current study is the first study to investigate the effects of 6-weeks of krill oil supplementation on exercise performance, markers of immune function and lipid peroxidation, and has shown that krill oil can increase PBMC IL-2 production and NK cell cytotoxic activity 3h post-exercise in both healthy young males and females without modifying performance. It is worth noting at this point that ideally we would have taking blood samples not only during the final time trial but also during the initial time trial, allowing a more direct comparison of responses in immune measures after the supplementation period in the same individuals, this is a limitation of the current study. It also remains to be established whether these alterations in immune function can ultimately reduce the burden of upper respiratory tract infections which are elevated in those with high training loads [44]. In that vein, our recent work has found that a supplement containing EPA, DHA, protein and vitamin D can reduce URTI symptoms, while not URTI numbers or duration, in active young males and females (3), although whether these effects are specifically due to EPA and DHA is not clear.
